# Violacein as a genetically-controlled, enzymatically amplified and photobleaching-resistant chromophore for optoacoustic bacterial imaging

**DOI:** 10.1038/srep11048

**Published:** 2015-06-19

**Authors:** Yuanyuan Jiang, Felix Sigmund, Josefine Reber, Xosé Luís Deán-Ben, Sarah Glasl, Moritz Kneipp, Héctor Estrada, Daniel Razansky, Vasilis Ntziachristos, Gil G. Westmeyer

**Affiliations:** 1 Institute for Biological and Medical Imaging (IBMI), Helmholtz Zentrum München, Neuherberg, Germany; 2 Institute of Developmental Genetics (IDG), Helmholtz Zentrum München, Neuherberg, Germany; 3 Chair for Biological Imaging, Technische Universität München (TUM), Munich, Germany; 4 Department of Nuclear Medicine, Technische Universität München (TUM), Munich, Germany

## Abstract

There is growing interest in genetically expressed reporters for *in vivo* studies of bacterial colonization in the context of infectious disease research, studies of the bacterial microbiome or cancer imaging and treatment. To empower non-invasive high-resolution bacterial tracking with deep tissue penetration, we herein use the genetically controlled biosynthesis of the deep-purple pigment Violacein as a photobleaching-resistant chromophore label for *in vivo* optoacoustic (photoacoustic) imaging in the near-infrared range. We demonstrate that Violacein-producing bacteria can be imaged with high contrast-to-noise in strongly vascularized xenografted murine tumors and further observe that Violacein shows anti-tumoral activity. Our experiments thus identify Violacein as a robust bacterial label for non-invasive optoacoustic imaging with high potential for basic research and future theranostic applications in bacterial tumor targeting.

Optoacoustic imaging comes with a strong potential for non-invasive cell-fate tracking, by enabling high-resolution cell visualization inside living tissues much deeper than what is possible with optical microscopy^1^. To enable optoacoustic cell detection with high sensitivity, labeling agents with a high molar absorbance (extinction) coefficient, low quantum yield and minimum photobleaching are desired^2^. For *in vivo* studies, genetic encoding of reporter chromophores may be superior to labeling approaches using synthetic dyes as it avoids signal loss due to serial dilutions of the contrast agent during cellular divisions.

Imaging bacterial populations in entire living host organisms is of increasing interest for infectious disease research^3^, studies of the microbiome^4^, as well as for theranostic applications in cancer research based on bacterial tumor targeting^5^,^6^. The latter approach relies on the preferential clonal expansion of bioengineered bacteria in the nutrient-rich, anaerobic and immunocompromised tumor microenvironment. Bacterial localization and tumor colonization can be determined by detecting reporter gene expression in targeted bacteria. So far, the luciferin-luciferase system^7^,^8^ ferritin^9^, magnetotactic bacteria^10^, or thymidine kinase^11^ have been employed as gene reporters for *in vivo* detection of bacterial colonization via bioluminescence, MRI, and PET imaging respectively. As for optoacoustic readout, point measurements of circulating bacteria tagged with nanoparticle-conjugated antibodies have been performed within blood vessels^12^. However, robust detection of genetically labeled bacteria *in vivo* via optoacoustic imaging has so far not been accomplished. One reason for this may be that common fluorescent proteins or chromoproteins often exhibit poor photostability making it challenging to obtain robust signals in optoacoustic imaging applications^13^. In contrast, enzymatically generated biosynthetic pigments such as melanin have the advantage of signal amplification because each genetically expressed enzyme can turn over many substrates per unit time. Although melanin produced in tyrosinase-overexpressing eukaryotic cells can be imaged by optoacoustics^14^,^15^, this approach has not yet been successfully transferred to bacterial optoacoustic imaging. In addition to melanin, other biosynthetic pigments such as riboflavin, canthaxanthin, carotenoids or Violacein (Vio) have been expressed in bacterial hosts for simple color differentiation by visual inspection^16^,^17^. The deep violet chromophore Violacein is of particular interest for detection in tissue as it has an absorbance spectrum peaking around 590 nm with substantial absorbance above 650 nm. Vio is enzymatically generated from the sole precursor tryptophan by five enzymes (VioA-E) that have originally been cloned from *Chromobacterium violaceum*^18^. While there may be a putative protective function of the violet pigment against visible radiation, Vio has also been reported to exert anti-protozoal and anti-tumoral activity against several tumor types^19^,^20^,^21^,^22^.

In search for a potent bacterial label that affords high-resolution, three dimensional bacterial imaging in tissues, we characterized the photophysical and biochemical properties of Vio with respect to optoacoustic detection and interrogated its performance as a bacterial label for optoacoustic imaging compared to melanin or fluorescent proteins. We further performed *in vivo* studies to characterize the capacity to detect Vio-labeled bacteria by multispectral optoacoustic tomography (MSOT) in tumor-bearing mice.

## Results

**Figure 1:**
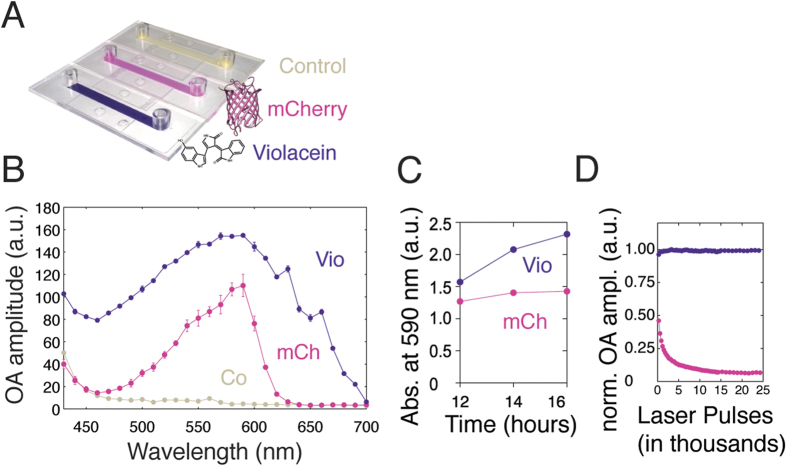
Comparison of photophysical parameters of Violacein-producing (Vio) and mCherry-expressing (mCh) bacteria. (**A**) Photograph of flow chips filled with bacterial suspensions producing the pigment Violacein (chemical structure shown), mCherry (protein structure shown) or control bacteria. (**B**) Optoacoustic spectra for both chromophore-containing bacterial strains and control bacteria (Co). (**C**) Time profile of chromophore production as measured by the absorbance increase at 590 nm. (**D**) Kinetics of photobleaching upon laser illumination at 590 nm.

We grew cultures of *E. coli* expressing the Violacein operon encoding the essential set of five enzymes (VioA-E) in the biosynthetic pathway for the production of Vio. As a reference chromophore we expressed the common fluorescent protein mCherry because of its comparable absorbance spectra and its prior use in optoacoustic imaging^23^. To compare the optoacoustic spectra of Vio and mCherry, we loaded cell culture flow chips with bacterial solutions of equal density (Fig. 1A) and placed them into a custom-built optoacoustic spectrometer connected to a tunable visible laser^24^. Vio-expressing bacteria exhibited a peak optoacoustic signal at ~590 nm with substantial signal still measurable above 650 nm (Fig. 1B), while mCherry expressing bacteria showed a slightly narrower optoacoustic spectrum peaking at 590 nm (absorbance peaks corresponded to optoacoustic signal maxima).

We subsequently measured the kinetics of Vio pigment formation in comparison to cells overexpressing tyrosinase, the rate-limiting enzymatic step for melanin synthesis. We thus serially sampled from culture flasks of *E. coli* expressing the respective chromophore and plotted the absorbance at 590 nm. Whereas the absorbance of mCherry plateaued around 16 hours after inoculation, Vio expressing cells reached a 1.6 fold higher value in absorbance with a continuing upward trend (Fig. 1C). In comparison, no substantial absorbance increase could be measured from the tyrosinase overexpressing cells grown in a shaking incubator; considerable melanin production could only be observed when bacteria were grown on agar plates supplemented with copper and L-tyrosine for a minimum of 48 hours (data not shown).

We were next interested in assessing the resistance of the chromophores against photobleaching, a critical requirement for obtaining robust signals in optoacoustic imaging. We first measured photobleaching with the optoacoustic spectrometer at 590 nm (Fig. 1D). Whereas mCherry expressing bacteria photobleached with a time constant of ~2150 pulses (95% confidence interval: 1940 to 2401), Vio-producing bacteria did not show significant bleaching after 25 thousand pulses *i.e.* over a duration of ~8 minutes.

The strong differences in bleaching rates have important consequences for the detectability of the different chromophores in optoacoustic microscopy, which achieves high spatial resolution but requires relatively high laser fluence^25^.

**Figure 2:**
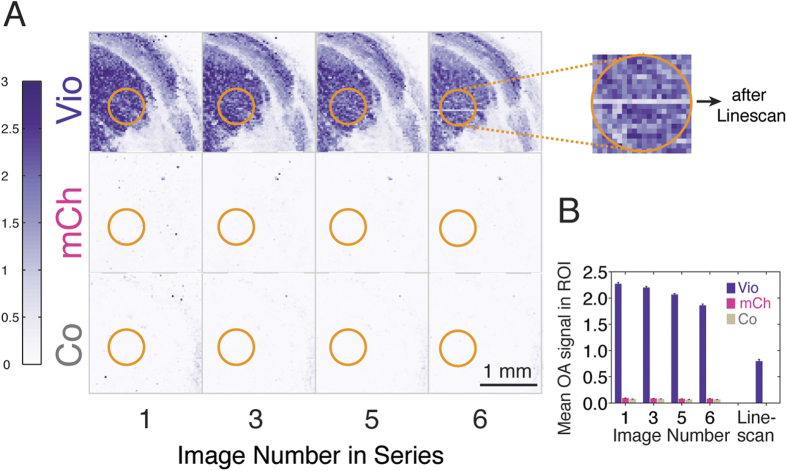
Optoacoustic microscopy images of bacterial suspensions. (**A**) Bacterial suspensions producing Violacein (Vio) or mCherry (mCh) and non-expressing controls (Co) were filled in circular wells of an agar phantom (~4 mm diameter, only upper right quadrant is shown) and sequentially imaged to assess image contrast and photobleaching. Repeated linescans were acquired through the well containing bacteria expressing Vio (magnification). (**B**) Optoacoustic signals for the three bacterial suspensions were averaged over the circular ROIs indicated in the figure (orange) at each time point. To assess the photobleaching after the line scan, all pixel intensities in the trajectory of the laser were averaged.

We filled ~4 mm diameter circular wells in an agar phantom with ~20 μL of the same bacterial solutions used to obtain the optoacoustic spectrum shown in Fig. 1B. The bacteria-containing agar phantom was then placed in an optoacoustic microscopy setup. Figure 2 displays images obtained from the upper right quadrant of the circular wells showing robust optoacoustic contrast from the Vio producing cells in repeated images as quantified by signal averaging over the indicated ROIs (Fig. 2B). In contrast, the signal from mCherry bleached very rapidly under the focused illumination of the microscopy system such that the signal averaged over the ROI indicated in the image (Fig. 2A) only marginally exceeded that of non-expressing control cells (Fig. 2B). This is remarkable since the identical sample of mCherry measured in the optoacoustic spectrometer on the same day yielded a signal that was 30% of that of Vio (Fig. 1B). To further quantify photobleaching of Vio-containing bacteria, we repeated a line scan through the top well over one minute (magnification in Fig. 2A) to deposit additional ~60 thousand pulses, resulting in a reduction of the signal to approximately 35% of that from the same region in the first image. When focusing the laser on a single 20 μm spot of the sample (~15 μJ average per-pulse energy), bleaching of Vio occured with a time constant of about 670 pulses (95% confidence interval: 664 to 675) while bleaching of mCherry happened so rapidly that the optoacoustic signal (averaged over the first 10 pulses) was only ~1.5 times higher than that of non-expressing control bacteria and only 1% of that expected from measurements of the identical sample on the optoacoustic spectrometer (Fig. 1B). In an independent experiment, we also compared the photobleaching rate of melanin-producing bacteria scraped off from confluent agar plates after 48 hours of growth (no substantial melanin production was observed in cultures grown in a shaking incubator). The resulting time constant for bleaching of the melanin-containing bacteria (imaging data not shown) was about 217 pulses (95% confidence interval: 215 to 219 pulses) and was slightly shorter than that for an equally small volume of Vio (264 pulses; 95% confidence interval: 261 to 268).

After characterizing the optoacoustic properties of Vio-expressing bacteria *in vitro*, we subsequently studied how well they can be detected in tumor-bearing mice by volumetric multispectral optoacoustic tomography (MSOT). We thus injected Vio-expressing (or non-expressing) bacterial suspensions into tumors grown from 4T1 mammary carcinoma cells xenografted a week prior to the imaging experiment.

**Figure 3:**
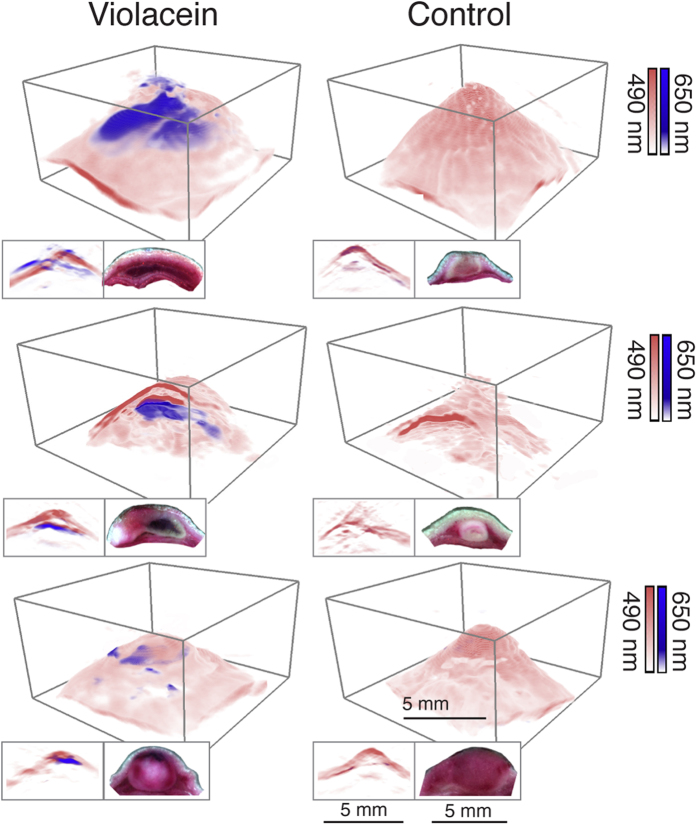
*In vivo* optoacoustic imaging of bacterially labeled 4T1 tumors in mice. Rendering of multispectral 3D optoacoustic data obtained from 4T1 tumors grafted in mice containing Violacein-expressing bacteria (first column) or non-expressing controls (second column). The white-to-blue and white-to-red color maps indicate the signal amplitudes of data acquired at 650 nm and 490 nm respectively. The insets underneath each 3D view show optoacoustic axial cross sections through the center of the volume (left) juxtaposed with color images of cryomicrotome sections through the *ex vivo* tumors (right).

We subsequently acquired multispectral optoacoustic images from the tumors with a portable three-dimensional optoacoustic imaging system connected to a tunable laser in the visible range^26^. Figure 3 shows the rendered 3D views of three tumors injected with Vio-containing bacteria and three tumors injected with control bacteria together with 2D projections taken through the center of each tumor juxtaposed with the corresponding cryomicrotome slice (insets). Vio-expressing bacteria could be clearly localized within the tumor tissue from the imaging data acquired at 650 nm (white-blue color scale), a wavelength at which Vio still generates substantial optoacoustic signal while the absorbance from blood is strongly reduced. Anatomical contrast is provided by displaying the data acquired at 490 nm on a color scale ranging from white to red. The variable distribution of Vio across animals, probably due to different injection mechanics into the differently shaped subcutaneous tumors, is detected with good agreement between the optoacoustic imaging and photographs taken from *ex vivo* axial cryomicrotome sections. In the first tumor, the distribution of Vio was widespread reaching superficial layers as also detected by a commercial optoacoustic imaging system with 100 μm in plane resolution using a near-infrared laser at 680 nm (Supplementary Fig. 1A). In the second tumor, Vio was located deeper in the tissue (~2.5 mm from the surface) as visualized by the cutaway 3D view and the 2D slice reconstructed from the optoacoustic data and the corresponding histological section. In the third tumor, a slightly smaller amount of Vio or control bacteria was injected at multiple positions within the tumor. Vio-expressing and control bacteria could also be detected in the tumor by immunohistochemistry (supplementary Fig. 1B). These imaging data demonstrate that the strong absorption of Vio-producing bacteria close to the near-infrared window labels tumors with good depth-resolution and high contrast-to-noise ratio (268 ± 59, mean ±standard deviation) as compared with tumors filled with non-expressing controls (21 ± 6).

**Figure 4:**
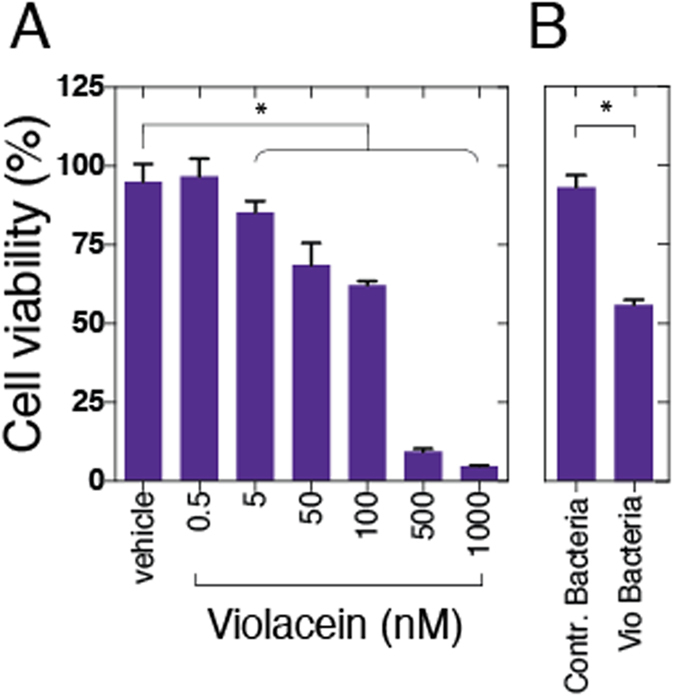
Effects of Violacein on cell viability of cultured 4T1 tumor cells. 4T1 cells were grown in 96 well plates, treated with nanomolar concentrations of pure Violacein (**A**) or Violacein released from bacteria (**B**) to compare cell viability against vehicle-treated cells (MTT assay).

With respect to possible future theranostic applications of Vio-expressing bacteria in bacterial tumor targeting, we also tested the anti-tumoral activity of purified Vio in a cell viability assay (MTT) on 4T1 tumor cells in culture (Fig. 4A). Significant reductions in tumor cell viability were observed at five nanomolar concentrations (one-way ANOVA with *p* < 0.0001; multiple comparisons against vehicle control with p < 0.05, Bonferroni corrected) with stronger effects measured after incubation with hundreds of nanomolar of Vio. We have furthermore assessed the effect of Vio released from bacteria via antibiotic treatment and found a significant effect (paired t-test, p < 0.0001) on 4T1 cell viability as compared to control bacteria (Fig. 4B).

This line of experiments thus jointly identifies Violacein as a photobleaching-resistant, high contrast-to-noise, genetically controlled and enzymatically amplified chromophore-label for non-invasive optoacoustic localization of bacteria in tumors of living mice with potential for future theranostic applications.

## Discussion

There is growing interest in longitudinal *in vivo* imaging of bacterial colonization as a method for determining the routes and bacterial burden of infections^27^,^28^,^29^, studying the microbiota^4^ and visualizing tumors via bacterial tumor targeting^8^. A general requirement for these imaging concepts is that the labeling agent is not diluted upon cellular divisions, which can occur at a fast rate in bacterial populations. This constitutes a strong advantage of genetically encoded contrast agents over synthetic labels as the genetic information is copied over to the progeny.

So far, mostly bioluminescent imaging has been used for bacterial tracking resulting in limited spatial resolution^7^. To enable three-dimensional imaging at much higher resolution via optoacoustics, we here focused on an enzymatically generated, photobleaching-resistant pigment and found it to be a potent bacterial label for optoacoustics. We were able to robustly detect ~6 × 10^9^ cfu bacteria in tumors, a number that is in the range of what was determined in tumor-tracking experiments after about one week of intratumoral bacterial growth post intravenous injection^6^,^30^.

With respect to alternative genetic labels for optoacoustic bacterial imaging, Vio has several advantages over common labeling strategies based on fluorescent proteins. First, the non-fluorescent pigment Vio is more efficient in converting the absorbed energy into thermoelastic expansion than fluorescent proteins that re-emit a portion of the absorbed photons. Second, even if chromoproteins optimized for low fluorescence are used^13^, each genetically expressed protein only harbors one chromophore, whereas many Vio pigments can be synthetized from the abundant substrate tryptophan by each instance of the enzymatic chain. This enzymatic amplification increases the sensitivity of bacterial detection, especially if only low protein expression levels can be achieved. Third, Vio is much more photobleaching-resistant than the common fluorescent protein mCherry. This advantage of the biosynthetic pigment likely carries over to chromoproteins with optimized photobleaching resistance as they were shown to exhibit a sharp signal decay when illuminated with three orders of magnitude lower fluence than was used here on the microscopic optoacoustic system^13^. In this regard it is interesting to investigate the maximum photostability that can be achieved with chromophores built from cyclized amino acids (as in *e.g.* proteins derived from Green Fluorescent Protein (GFP)) compared with chromophores bound to the protein backbone (such as biliverdin and infrared fluorescent protein (iRFP)^31^) or (bio)synthetic dyes.

With respect to the goal of non-invasive tracking of bacterial colonization, it is furthermore desired that the cellular contrast agent is generated with fast kinetics to match the rapid bacterial growth. Based on the data presented here, bacteria expressing the Violacein operon showed fast production of Vio that did not show the early saturation of the optoacoustic signal measured from the mCherry-expressing bacteria; this could be explained by the shorter life time of the fluorescent protein as compared with that of the pigment Vio. It is furthermore of interest that the multi-enzymatic biosynthesis of Vio outperformed melanin production from tyrosinase overexpressing cells. In our hands, those cells produced melanin only with supplementation of excess tyrosine and the cofactor copper and only when grown on agar plates for a minimum of 48 hours. Growth could however not be achieved in shaking cultures for even after 24 hours of incubation. This points to an advantage of Vio as a genetic bacterial label as all steps of its synthesis are enzymatically catalyzed and do not rely on spontaneous cyclization necessary for the maturation of many chromoproteins or polymerization reactions as in the case of melanin formation.

In summary, we have shown that Vio is an excellent biosynthetic contrast agent for optoacoustic bacterial detection *in vivo* that very favorably compares with a common fluorescent protein (mCherry) or the biosynthetic pigment melanin. Vio generates a strong optoacoustic signal close to the near-infrared range and exhibits high photobleaching resistance. The bacterial production of Vio is enzymatically amplified, fast and robust. Vio-expressing bacteria can be differentiated well by multispectral optoacoustic imaging from strongly vascularized xenografted tumors in living mice. Vio’s distinct optoacoustic spectrum may also afford multiplexing applications for bacterial detection together with optimized chromoproteins or biosynthetic pigments such as melanin (if bacterial production of the latter can be optimized). The robust enzymatic amplification and fast pigment formation of the Violacein system also qualify it as an effective output of weaker promoters or genetic circuits that may be introduced to turn the bacterium into a whole-cell sensor for environmental parameters or analytes of interest^32^. In the context of bacterial tumor tracking, it may be of particular interest to couple Vio production to sensing of *e.g.* vascular growth factors or hypoxic conditions. With respect to an extension of this concept for future theranostic applications, it is also intriguing to study the anti-cancer activity of Violacein-expressing bacteria *in vivo* and investigate whether the release of Violacein could be coupled to genetic sensor circuits and/or placed under pharmacogenetic control^33^. Non-invasive control over Vio’s anti-tumoral activity may also be effectively achieved via antibiotic drugs or cell-permeabilization by laser illumination or highly focused ultrasound.

## Methods

### Genetic reporter constructs and bacterial growth

pVIO1-2 was created by Sánchez *et al.*^18^, by isolating the vio locus (VioA-E) from genomic DNA of *Chromobacterium violaceum* (ATCC12472) as an 8.9 kb MluI-XhoI fragment and subcloning it into lac promoter driven LITMUS 38 with MluI and SalI. MelA from *Rhizobium etli* was amplified from pTrc MelA (kindly provided by Dr. Guillermo Gosset Lagarda) and subcloned into pmCherry with AgeI and NotI. pVIO1-2 (kindly provided by Dr. José A. Salas), pmCherry (kind gift from Dr. Arie Geerlof, Helmholtz Zentrum München), pMelA and pUC19 (control bacteria) were transformed into TOP10 Chemically Competent *E. coli* (Invitrogen Carlsbad, CA, USA) according to the protocol of the manufacturer. Single colonies were picked and inoculated into 250 mL LB-medium containing 100 μg/mL ampicillin. Cultures were grown for 18 h at 37 °C and 250 rpm. Violacein-producing bacteria showed no statistically significant difference to control bacteria (Wilcoxon matched-Pairs Signed-Ranks Test) with respect to cell viability (BacTiter-Glo™luciferase assay) or cell wet weight. Plasmid retention was tested *in vitro* and showed that the bacteria retain the plasmid without selection pressure for at least 7 days without any significant loss of violacein production.

To yield bacterial suspensions, cultures were spun down at 4000 rpm for 15 min at 4 °C and resuspended in 25% (weight PBS/wet weight bacteria) in PBS. As no substantial melanin formation was observed from MelA expressing bacteria in liquid culture, MelA expressing bacteria were grown on LB agar plates supplemented with 2 mM L-tyrosine and 400 μM CuSO_4_ for 48 h. For photobleaching experiments, MelA expressing bacteria were scraped off the plates and resuspended in 50% (weight PBS/wet weight bacteria) PBS.

### Assessing chromophore maturation times

Single colonies of *E. coli* TOP10 cells transformed with pVIO1–2 or pmCherry were picked and inoculated into 250 mL LB medium containing 100 μg/mL ampicillin. At 12, 14 and 16 h of growth time, 50 mL of the bacterial cultures were spun down at 4000 rpm for 15 min at 4 °C and resuspended in 25% (weight PBS/wet weight bacteria) in PBS. For absorbance measurements, bacterial suspensions were diluted 1:5 in PBS and absorbance was measured at 590 nm on a SpectraMax M5 (Molecular Devices, Sunnyvale, USA).

### Measurement of optoacoustic spectra

Cell culture flow chips (μ-Slide I 0.2 Luer, hydrophobic, uncoated, sterile, IBIDI, Martinsried, Germany) were connected to plastic tubing and placed upside down into a sample holder fixed to the frame of a custom-built optoacoustic spectrometer. The flow channel’s thin transparent foil was placed in the acoustic focus of the ultrasound detector (V382-SU, 3.5 MHz Immersion Transducer, Olympus, Hamburg, Germany) mounted on the device’s plexiglas roof. The device was then placed resting on an optical table within a water basin to ensure acoustic coupling between the sample and the ultrasound detector. The output of a tunable visible laser (SpitLight DPSS 250, Innolas, Krailling, Germany) was guided onto the flow channel of the cell culture chip via mirrors. *E. coli* suspensions with weight-adjusted densities were sequentially injected into the flow channel of the cell culture chip via the tubing and optoacoustic signals were recorded (NI USB-5133, National Instruments, Austin, Texas, USA connected to a standard computer) as a function of laser wavelength. The flow channel was washed in between measurements with 10 channel volumes of PBS such that no optoacoustic signal remained. PBS was then pushed out of the channel by injecting air to ensure that no dilution or inhomogeneity of subsequent samples occurred. A laser power meter (FieldMaxII-TOP, Coherent Inc. Santa Clara, USA) was used to measure the laser power as a function of wavelength to normalize the optoacoustic signal amplitude for each wavelength applying custom routines implemented in MATLAB (Mathworks, Natick, USA). Absorbance measurements were taken on a plate reader (SpextraMax M5, Molecular Devices, Sunnyvale, USA). Spectra are shown as mean values with error bars representing the standard error of the mean. Photobleaching for these illumination conditions was assessed by repeated measurements at 590 nm for Vio. Curves were plotted and fitted with a monophasic exponential decay function or linear regression using GraphPad Prism 6 (GraphPad, La Jolla, USA). Figures were prepared in Adobe Illustrator. The mCherry structure shown as inset in Fig. 1 A was generated from PDB entry 2H5Q using UCSF Chimera.

### Optoacoustic *in vitro* microscopy

Bacterial suspensions (20 μL of the same suspension from which optoacoustic spectra were obtained) were filled into an agar phantom (1.3% agarose in PBS) containing three circular wells of ~4 mm diameter and positioned under the motorized stage-mounted coaxial optoacoustic head of an optoacoustic microscope adapted from Estrada *et al.*^24^. The optical excitation was provided by a pump laser Nd:YAG Q-switched laser (model IS8II-E, Edge- Wave GmbH, Wuerselen, Germany) feeding a dye laser (Credo, Sirah Lasertechnik GmbH, Grevenbroich, Germany) loaded with Pyrromethene 597 dye (maximum wavelength conversion efficiency at 585 nm) tuned to 594 nm. By means of a Gradient-Index (GRIN) lens assembly, the system generated a diffraction-limited 20 μm diameter (FWHM) optical focus at the acoustic focus of the 30 MHz polyvinylidene difluoride (PVdF) ultrasound detector (Precision Acoustics, Dorchester, United Kingdom). The average per-pulse energy was maintained at ~15 μJ. Thus high fluence in excess of 2 J/cm^2^ were generated at the sample surface. Sequential point measurements were taken over time at the center of the respective sample well (over 15 seconds at 5 kHz laser repetition rate) to measure signal decay due to photobleaching under focused illumination conditions commonly used in optoacoustic microscopy systems. Signal curves were fitted to a monophasic exponential decay function with GraphPad Prism. Subsequently, five spatially resolved optoacoustic measurements were taken repeatedly with both the optical beam and ultrasonic detector quickly scanned across the sample using step-size of 30 μm to assess signal amplitude and photobleaching. Image volumes were reconstructed using custom-written routines^24^ implemented in MATLAB and displayed as 2D maximum amplitude projections. Mean optoacoustic signal amplitude were averaged from disk-shaped ROIs with a diameter of 600 μm (error bars represent the standard error of the mean). To further assess the kinetics of photobleaching of Vio, horizontal line scans were taken through the well containing Vio with a pulse repetition frequency of 1 kHz over 1 min in total, before an additional image of all sample wells was acquired.

### *In vivo* imaging by means of volumetric multispectral optoacoustic tomography (MSOT)

8-10 week-old nude mice (matched pairs of Foxn1 - CD1 (f) and Balb/c (m); Harlan Laboratories, Germany) were inoculated with 4T1 cells (1 × 10^6^ cells in 30 μL of PBS) into the subcutis of the neck. After 7 days of tumor growth, animals were deeply anesthetized, placed on a heat-blanket and a total 20 μL of Vio-expressing *E. coli* or control bacteria (~0.5 × 10^9 ^cfu/μL, 0.3 × 10^9^ cfu/μL for the pair of datasets displayed on the bottom of Fig. 3) were intratumorally injected at multiple locations within each tumor. Animals were subsequently positioned in supine orientation on a portable volumetric optoacoustic imaging system^25^ with acoustic matching provided by optically transparent agar. The system was coupled to a tunable visible laser (SpitLight DPSS 250, Innolas, Krailling, Germany), which provided short-duration (<10 ns) laser pulses with optical wavelengths tuned between 450 to 650 nm in 10 nm increments. Optoacoustic image volumes were acquired and rendered in real time using model-based three-dimensional reconstruction algorithm, essentially retrieving the volumetric distribution of optical absorption at each wavelength with almost isotropic resolution of 200 micrometers^34^. Contrast-to-noise ratios (CNRs) were computed by placing a cubical ROI (0.027 mm^3^) into the tumor, the skin close to the edge of the FOV and background outside of the tissue and taking the absolute difference of signals acquired at 650 nm from tumor and skin divided by the standard deviation of the background noise. The imaging data shown in supplementary Fig. 1 was acquired on an inVision 256-TF system (iThera Medical, Munich, Germany) at 680 nm and 710 nm. All animal experiments were approved by the government of Upper Bavaria and were carried out in accordance with the approved guidelines.

### *Ex vivo* sectioning on cyromicrotome

After completion of imaging, the mice were euthanized with a lethal dose of ketamine/xylazine and frozen to −20 °C. The upper torso including the tumor was embedded in O.C.T (TissueTeck). The embedded tissue was cryosliced along the axial planes every 50 micrometers in a modified Leica cryotome combined with a CCD camera to capture RGB color images from the surface on the bulked remaining sample. For bacterial detection, tissue sections were equilibrated to room temperature and rehydrated in PBS for 10 min and blocked in 3% BSA PBS for 30 minutes at room temperature. Subsequently, tissue sections were incubated with Escherichia coli BioParticles® Opsonizing Reagent (Molecular Probes) diluted 1:100 in PBS for 30 minutes at room temperature. After incubation, slices were washed three times with PBS. Subsequently, slices were incubated with 4 μg/mL goat-anti-rabbit DyLight594 (Abcam ab96897) antibody conjugate for 30 minutes at room temperatures and then washed three times with PBS. Microscopy of the tissue sections was performed on an Olympus IX81 confocal microscope.

### Cell viability assay

The 4T1 cell line, a mouse mammary tumor cell line (CRL-2539; American Type Cell Culture Collection, Manassas VA), was grown in 5% CO_2_ at 37 °C in RPMI-1640 medium (Sigma-Aldrich) containing 10% fetal bovine serum and antibiotics (penicillin and streptomycin). Routine culture treatment was conducted twice a week. Cells were harvested and seeded in 96-well plates at 2.5 × 10^3^ cell per well in a final volume of 100 μL. After overnight incubation for cell adhesion, the medium was removed, and the cells were incubated at 37 °C with 100 μL of medium containing Vio (0.5 nM–1.0 μM; Vio from *Janthinobacterium lividum*, Sigma-Aldrich), 10% dimethyl sulfoxide (DMSO) as a negative control, 0.1% DMSO as vehicle control of Vio and RPMI medium only to define 100% viable cells. After 24 h of incubation, the medium containing the tested compound was removed and fresh medium was added. Cell viability was determined by a colorimetric cell viability assay (Cell Proliferation Kit 1 (MTT), Roche) used according to the manufacturer’s instructions. The absorbance measurement was performed on a microplate reader (Infinite® 200 PRO, Tecan) and was determined at 595 nm, whereby, 651 nm was used as a reference wavelength. To quantify cell viability, the ratio of the absorbance of the samples to the absorbance of the non-treated control samples (=100%) was calculated (the bargraph shows the mean, error bars have the length of one standard deviation). To determine anti-tumoral activity of Vio released from bacteria, Vio-producing and control bacteria were grown for 18 h at 37 °C, which yielded cultures containing around 5 × 10^9 cfu/mL and approximately 10 μM of Vio as determined photospectrometrically after aceton extraction (using an extinction coefficient of 32900 M^−1^ cm^−1^@ 555 nm). Bacteria were spun down, the supernatant was discarded and the bacteria were resuspended in an equal volume of PBS. In order lyse the bacteria, kanamycin solution was added to yield a final concentration of 50 μg/mL. After, bacteria were incubated for 2 d at RT to achieve complete lysis. Subsequently, bacterial lysates were diluted 1:10 in cell culture medium to yield a concentration of released Vio of approximately 1 μM and used for the MTT assay.

## Additional Information

**How to cite this article**: Jiang, Y. *et al.* Violacein as a genetically-controlled, enzymatically amplified and photobleaching-resistant chromophore for optoacoustic bacterial imaging. *Sci. Rep.* **5**, 11048; doi: 10.1038/srep11048 (2015).

## Supplementary Material

Supplementary Information
