# Supplementary material for: Violacein as a genetically-controlled, enzymatically amplified and photobleaching-resistant chromophore for optoacoustic bacterial imaging
Source: Sci Rep. 2015 Jun 19;5:11048. doi: 10.1038/srep11048 (PMC4473533; doi:10.1038/srep11048)
Supplement: Supplementary Information [file srep11048-s1.doc]

Supplementary information

Violacein as a genetically-controlled, enzymatically amplified and photobleaching-resistant chromophore for optoacoustic bacterial imaging

**Yuanyuan Jiang^1,2+^, Felix Sigmund^1,2+^, Josefine Reber^1^ , X. Luís Deán-Ben^1^, Sarah Glasl^1^, Moritz Kneipp^1,3^, Héctor Estrada^1^, Daniel Razansky^1,3^ Vasilis Ntziachristos^1,3^ & Gil G. Westmeyer^1,2,4^**^*^

*^1^Institute for Biological and Medical Imaging (IBMI), Helmholtz Zentrum München, Neuherberg, Germany*

*^2^Institute of Developmental Genetics (IDG), Helmholtz Zentrum München, Neuherberg, Germany*

*^3^Chair for Biological Imaging and ^4^Department of Nuclear Medicine, Technische Universität München (TUM), Munich, Germany*

*+these authors contributed equally to this work*

^*^Corresponding author: *gil.westmeyer@tum.de*


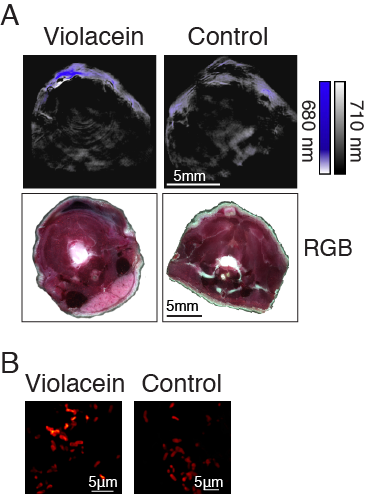


Suppl. Fig. 1. **Optoacoustic Imaging with NIR laser and Immunohistochemical detection of bacteria.** The tumor shown in the upper left panel of Fig. 3 was also imaged on an inVision 256-TF small animal scanner coupled to a NIR laser (**A**). Fluorescence microscopy images of Vio-expression and control bacteria visualized after immunohistochemical detection on fixed cryomicrotome slices (**B**).
